# Regulation of PD-1/PD-L1 pathway and resistance to PD-1/PD-L1 blockade

**DOI:** 10.18632/oncotarget.22690

**Published:** 2017-11-25

**Authors:** Jie Bai, Zhitao Gao, Xiang Li, Liang Dong, Weidong Han, Jing Nie

**Affiliations:** ^1^ Department of Molecular Biology and Bio-Therapeutic, School of Life Science, Chinese PLA General Hospital, Beijing 100853, China

**Keywords:** PD-1, PD-L1, resistance, epigenetic, tumor microenvironment

## Abstract

Immune checkpoint blockades, such as inhibitors against programmed death 1 (PD-1) and its ligand (PD-L1), have received extensive attention in the past decade because of their dramatic clinical outcomes in advanced malignancies. However, both primary and acquired resistance becomes one of the major obstacles, which greatly limits the long-lasting effects and wide application of PD-1/PD-L1 blockade therapy. PD-1/PD-L1 both regulates and is regulated by cellular signaling pathways and epigenetic modification, thus inhibiting the proliferation and effector function of T and B cells. The lack of tumor antigens and effective antigen presentation, aberrant activation of oncogenic pathways, mutations in IFN-γ signaling, immunosuppressive tumor microenvironment such as regulatory T cells, myeloid-derived suppressor cells, M2 macrophages, and immunoinhibitory cytokines can lead to resistance to PD-1/PD-L1 blockade. In this review, we describe PD-1 related signaling pathways, essential factors contributing to the resistance of PD-1 blockade, and discuss strategies to increase the efficacy of immunotherapy. Furthermore, we discuss the possibility of combined epigenetic therapy with PD-1 blockade as a potential promising approach for cancer treatment.

## INTRODUCTION

The “two-signal theory” of lymphocyte activation explains the mechanism of T cell activation or anergy when a naive T cell makes contact with an antigen [1, 2]. Accordingly, efficient activation of antigen-specific lymphocytes needs specific antigen recognition by lymphocytes and an additional signal. Later, it was found that coinhibitory signals also exist. Receptors eliciting coinhibitory signals function as immune checkpoints and have an essential status in the maintenance of peripheral tolerance and inhibition of autoimmunity [3–6].

The best-studied pathway of T-cell costimulation involves the B7-CD28-CTLA-4 superfamily, of which PD-1 and its ligands belong, revealed that the immune system has developed several coinhibitory pathways for T cell activation and tolerance [7–11]. Costimulation has been of therapeutic interest in cancer therapy because the augmentation of costimulatory signals could promote T cell activation to enhance antitumor immune responses [12]. With the discovery of CTLA-4 as an effective immune checkpoint, blockade of CTLA-4 was found to promote antitumor immune reactions and gain notable clinical effectiveness as cancer therapy [13, 14]. The understanding of cancer immunotherapy was modified and strategies for removing other coinhibitory signals to activate the immune system were widely investigated. The tumor immunotherapy targeting PD-1/PD-L1 has achieved encouraging therapeutic outcomes, with response rates of 20% to 40% in various cancer types [15]. Thus far, five immune checkpoint inhibitors targeting PD-1/PD-L1 have been approved by the US Food and Drug Administration, such as PD-1-blocking monoclonal antibodies (mAb) pembrolizumab and nivolumab, and PD-L1-targeted mAb atezolizumab, avelumab, and durvalumab. Compared with anti-CTLA-4 mAb, immune-related toxicities induced by PD-1/PD-L1 blockade were much less frequent and the most frequently observed toxicity was fatigue [16, 17].

## PD-1 PATHWAY

### Expression of PD-1 and PD-1 ligands

PD-1 is expressed on activated CD4 and CD8 T cells, B cells, monocytes, natural killer (NK) cells, and dendritic cells (DCs) [18–21]. The expression of PD-1 on T cells can be induced by the common γ chain cytokines interleukin-2 (IL-2), IL-7, IL-15, and IL-21 [22]. PD-1 is encoded in the *Pdcd1* gene. Nuclear factor of activated T-cells cytoplasmic 1 (NFATc1) is a significant transcription factor, which promotes the PD-1 expression [23]. Other established transcriptional activators such as forkhead box O 1 (Foxo1), Notch proteins, and interferon regulatory factor 9 (IRF9) can also promote PD-1 transcription, and T-box transcription factor TBX21 (T-bet) functions as a transcriptional repressor [24–27]. During chronic viral infection, PD-1 expression is enhanced and maintained on exhausted virus-specific T cells to prevent their proliferation and function [28, 29]. CpG oligodeoxynucleotides treatment induced PD-1 expression in human CD19+ B cells [30]. Environmental hyaluronan fragments from hepatoma cells produced PD-1^high^ regulatory B cells via TLR4 activation, during which TLR4-mediated Bcl-6 upregulation was critical [31]. Interferon (IFN)-sensitive responsive element (ISRE) and STAT1/2 regulate PD-1 expression mediated by IFN-γ in macrophages [32].

PD-L1 (B7-H1 or CD274) and PD-L2 (B7-DC or CD273) are the ligands of PD-1, which are type I transmembrane glycoproteins. There is approximately 40% of the same acidic identity between PD-L1 and PD-L2 whereas the similarity between PD-Ls and B7s is 20% [33, 34]. PD-Ls have different patterns of expression. The expression of PD-L1 constitutively exists on T and B cells, DCs, macrophages, mesenchymal stem cells, and bone marrow-derived mast cells [19]. PD-L1 is also expressed on a large-scale in nonhematopoietic cells such as lung, vascular endothelial, fibroblastic reticular, liver nonparenchymal, and mesenchymal stem cells, and pancreatic islets, astrocytes, neurons, and keratinocytes [20]. In contrast with PD-L1, PD-L2 expression is restricted to activated DCs, macrophages, bone marrow-derived mast cells, and over 50% of peritoneal B1 cells [35]. PD-L1 expression can be induced by γ chain cytokines IL-2, IL-7, and IL-15 on T cells, and IL-21 promoted PD-L1 expression on CD19+ B cells. LPS or BCR activation also stimulate the expression of PD-Ls on B cells [36–38]. Treatment of interferon-gamma (IFN-γ) or IL-10 results in the expression of both ligands in monocytes, and IL-4 and granulocyte macrophage colony-stimulating factor (GM-CSF) induce PD-L2 expression on DCs [39]. In tumor cells, the PD-1 and PD-1 ligands ligation mediates inhibitory signals to cause a harmful effect on antitumor immunity, resulting in the escape from immunosurveillance [40–42].

### The influence of epigenetic modification on PD-1 expression

Epigenetic modification, including DNA methylation, histone methylation/acetylation, and microRNA regulation, also controls the expression of PD-1. During acute infection, CD8+ T cell differentiation from naïve T cells was accompanied by transient DNA demethylation at *Pdcd1* locus, which gained DNA methylation during further differentiation into functional memory T cells. In contrast, PD-1 promoter was dramatically demethylated in exhausted CD8+ T cells and imprinted during the effector phase of CD8 T cell exhaustion [43, 44]. In patients with chronic HIV, PD-1 promoter was demethylated in PD-1-high virus-specific T cells, and methylated in naïve, PD-1-low T cells from the same donors, while after anti-retroviral therapy, there is no remethylation of DNA at the PD-1 promoter, indicating that the poised epigenetic status for PD-1 remained after prolonged exposure to HIV virus [45]. Modifications to histone proteins also lead to changes in *Pdcd1* transcription. Enhancers are marked by histone H3 lysine 4 monomethylation (H3K4me1) and H3K27 acetylation (ac) at the “active” status [46], and other activation marks such as H3K9ac and H3K27ac were enriched at the promoter when PD-1 expression was induced on CD8 T cells *in vitro* [47, 48]. MicroRNAs also take a part in PD-1 expression. In the melanoma-bearing mice, the expression of PD-1 was attenuated after transfection with miR-28 mimic [49]. In immunocompetent murine models, miR-138 treatment of GL261 gliomas reduced the expression of PD-1, CTLA-4, and FoxP3 in T cells, promoting tumor regression [50]. Moreover, in lymphocytes from patients with chronic hepatitis B virus, transfection of miR-4717 mimics significantly decreased PD-1 expression in concert with increased tumor necrosis factor-α (TNF-α) and IFN-γ production [51].

### The PD-1 downstream signaling pathway

The mechanism of how PD-1 inhibits T-cell receptor signaling is a focus of investigation. Beginning with recruiting SHP-2 (SRC Homology 2-Domain-Containingprotein Tyrosine Phosphatase 2) proximate to the T-cell receptor, PD-1 ligation inhibits the activation of T-cell receptor proximal kinases, resulting in depression of the phosphorylation of ZAP-70 mediated by Lck and initiation of downstream events [52].

The PI3K/Akt signaling is an important target of the PD-1 downstream pathway [53]. First, PD-1 can inhibit the activation of PI3K by recruiting SHP-2 [54]. As a serine-threonine phosphatase, PTEN inhibits the activation of PI3K and suppresses the PI3K-Akt pathway signal transmission mediated by CK2. During T-cell activation, PTEN is phosphorylated by CK2 in the S380-T382-T383 cluster, resulting in PTEN stability and reducing PTEN phosphatase activity. PD-1 can target and inhibit CK2-mediated PTEN phosphorylation and promote its degradation while it induces PTEN phosphatase activity, thus repressing PI3K/Akt signaling [55–57].

The Ras/MEK/ERK pathway is another signaling pathway regulated by PD-1 [52, 53]. In T cells, the activation of RasGRP1 is critical for the activation of Ras and downstream MEK/ERK MAP kinase, and RasGRP1 is activated by calcium and diacylglycerol downstream of PLC-γ1 [58]. The activation of PLC-γ1 and Ras are inhibited by PD-1, resulting in diminished activation of the MEK/ERK pathway [53].

By similar mechanisms in B cells, PD-1 ligation with B-cell receptor (BCR) signaling results in SHP-2 recruitment to the ITSM tyrosine of PD-1, and inhibits BCR-mediated Ca^2+^ mobilization and tyrosine phosphorylation of effector molecules, including Ig β, Syk, phospholipase C-γ2 (PLC- 2), and ERK1/2 [59].

## RESISTANCE TO PD-1/PD-L1 BLOCKADE

Immunotherapy has been viewed as one of the most promising methods for cancer treatment. Blocking of PD-1/PD-L1 interaction could overcome the counteraction and preserve the antitumor capacity of T cells to repress tumor cells [14, 15, 60]. Thus far, five PD-1/PD-L1 blocking mAbs have been approved by the U.S. FDA, and the superiority has been confirmed in over 15 cancer types [61]. Nevertheless, as compared to chemotherapy and molecular targeted therapy, a relatively higher rate of primary resistance with immune checkpoint inhibitors occurs and depresses the effectual clinical benefits. The efficacy of monotherapy for PD-1/PD-L1 blockade was rarely more than 40%, with a large proportion of partial responders [15, 62]. Moreover, after an initial response to PD-1/PD-L1 blockade, acquired resistance occurs in most patients, which leads to disease progression or relapse eventually.

**Figure 1:**
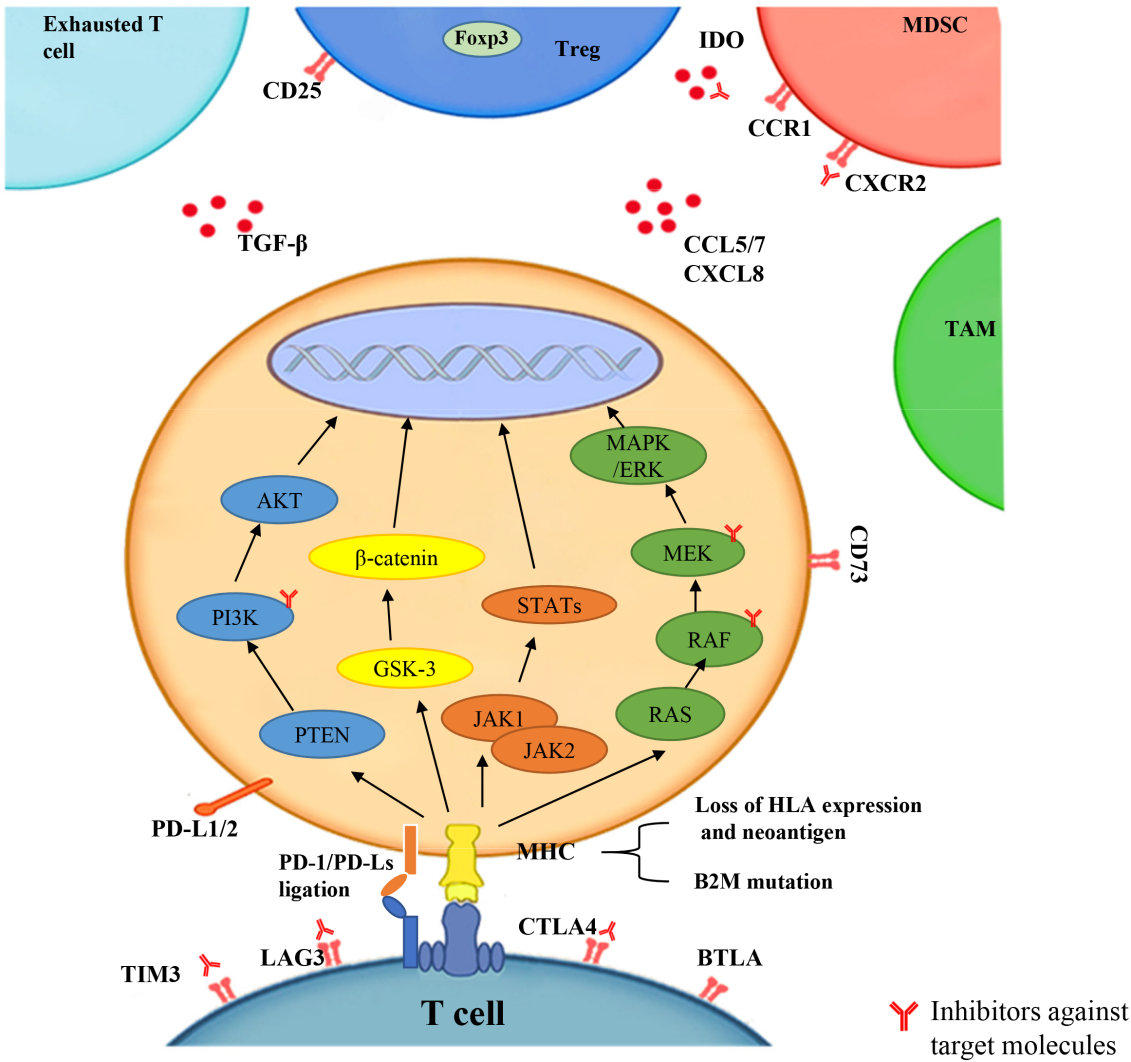
The factors that lead to resistance to PD-1 blockade include PD-L1 expression, tumor neoantigens expression and presentation, cellular signaling pathways (PI3K, WNT, IFN-γ, MAPK), tumor microenvironment (TME) (exhausted T cell, Treg, MDSC, TAM, other chemokines), and related immune genes (IPRES) The inhibitors against target molecules are indicated, which could enhance antitumor responses in in mouse models when combined with PD-1/PD-L1 blockade.

The mechanisms of resistance are complex, dynamic, and interdependent. There are many tumor cell-intrinsic and -extrinsic factors relating to PD-1 blockade resistance, including PD-L1 expression, tumor neoantigen expression and presentation, associated cellular signaling pathways, tumor microenvironment (TME), related immune genes, and epigenetic modification (Table 1 and Figure 1).

**Table 1:** The mechanism of resistance to PD-1/PD-L1 blockade

Mechanism	Description	Reference
**PD-L1 expression**	PD-L1/2 expression could be associated with the higher local immune cytolytic activity and clinical response.	[63–69]
**Lack of effective antigen presentation**	The most straightforward reason why tumors would not respond to PD-1/PD-L1 blockade therapy is lack of recognition by T cells via the mechanisms such as absence of tumor antigens, loss of HLA expression, and Dysfunctional mutations in B2M.	[15, 70–78]
**Cellular signaling pathways**		
PI3K/AKT pathway	Loss of PTEN-mediated PI3K/AKT activation significantly correlated with the decreased expression of IFN-γ, granzyme B, less CD8 T cell infiltration.	[79]
WNT/β-catenin pathway	Stabilization of b-catenin resulting in constitutive WNT signaling pathway could induce T cell exclusion from cancers.	[80]
JAK/STAT/IFN-γ pathway	Cancer cells could escape the effects of IFN-γ by downregulating or mutating molecules including IFNGR1/2, JAK2, and IRF1.	[81–88]
MAPK pathway	With the inducement of VEGF and IL-8, MAPK signaling has inhibitory effects on T cell recruitment and function.	[89–92]
**Tumor microenvironment**		
Immunosuppressive cells		
Exhaustion T cells	Disfunctional T cells and PD-1^high^ phenotype exhaustion T cells cannot benefit from PD-1 blockade.	[94]
Tregs	Suppress effector T cell (Teff) responses by secretion of IL-10, IL-35, and TGF-β.	[95–101]
MDSCs	Promote angiogenesis, tumor invasion and metastasis.	[102–106]
TAMs	Higher frequencies of TAMs are associated with poor prognosis.	[41, 107–110]
Immunosuppressive cytokines	Often released by tumor or macrophages for local suppression of anti-tumor immune responses, including TGF-β, CCL5, CCL7, CXCL8, IDO, etc.	[111–118]
Inhibitory receptors	Apart from PD-1, over-expression of multiple inhibitory receptors including TIM3, CTLA4, LAG3 and BTLA is associated with inhibition of T-cell function and resistance to PD-1/PD-L1 blockade therapy.	[119, 120]
**Immune related genes**		
IPRES signatures	Within TME, co-enrichment of a group of 26 transcriptomic signatures (named IPRES signatures) was also related to primary resistance to PD-1/PD-L1 blockade.	[121]
**Epigenetic modification**		
DNA methylation and histone acetylation	Epigenetic modification may lead to changes in immune-related genes expression to impact antigen processing, presentation, immune evasion and T cell exhaustion, and DNA methyltransferase inhibitors and histone deacetylase inhibitors can reverseimmune suppression via several mechanisms.	[122–134]

### PD-L1 expression

In Hodgkin's lymphoma, frequent amplification of chromosome 9p24.1 that encodes PD-1 ligands PD-L1 and PD-L2 was observed, and the active JAK/STAT signaling further induced PD-L1/2 expression, which could be associated with the higher clinical response in Hodgkin lymphoma in response to PD-1/PD-L1 blockade [63]. In addition, genetic amplification of PD-L1/2 was positively correlated with high local immune cytolytic activity [64]. Other mechanisms to promote constitutive PD-L1 expression in cancer cells included PTEN deletions, PI3K and/or AKT mutations, EGFR mutations, MYC overexpression, CDK5 disruption, and an increase in PD-L1 transcripts stabilized by truncation of the 30 UTR of this gene [65–69]. It is currently undefined whether constitutive PD-L1 expression increases or reduces the sensitivity to PD-1/PD-L1 blockade therapy.

### Lack of tumor antigens and effective antigen presentation

The most straightforward reason why tumors would not respond to PD-1/PD-L1 blockade therapy is lack of recognition by T cells because of absence of tumor antigens [70]. Human melanoma, renal cell carcinoma (RCC), and non small cell lung cancer (NSCLC) are highly immunogenic mutations, ranging from 5 to 10 per mega-base of DNA in the range of individual cell mutations [15, 71]. This finding is consistent with the clinical results that these tumors are most sensitive to PD-1/PD-L1 blockade therapy [72, 73]. Poorly immunogenic tumors that tend to have between 0.1-1 somatic mutation per mega-base of DNA largely show no response to PD-1/PD-L1 blockade, such as in the pancreas and prostate [71]. Meanwhile, mutational load is also associated with efficacy in specific tumor types. In two groups of patients with NSCLCs, a higher burden of nonsynonymous somatic mutations in tumors was detected, which was related to better clinical response and longer survival [72].

Decrease in neoantigens is also an important mechanism for acquired resistance to immunotherapy. In T cell-dependent immunoselection, Elimination of neoantigens has been considered as a mechanism of cancer immunoediting in mice [74]. A recent study revealed that the evolution of neoantigen loss could augment acquired resistance as an escape mechanism after PD-1/PD-L1 blockade treatment. In the NSCLC relapsed patients after initial response, a loss of 7 to 18 putative mutation-associated neoantigens in resistant clones was observed in the analysis of protein coding genes of matched pretreatment and resistant cancers [75].

Moreover, cancer cells may also develop mechanisms to avoid antigen processing and presenting to the cell surface, via silencing or altering the expression of antigen-presenting machinery, beta-2-microglobulin (B2M), or MHC molecules [76, 77]. B2M plays an essential role in supporting MHC class I molecules to present tumor specific peptides to T cells. Dysfunctional mutations in B2M have been viewed as an important mechanism of tumor resistance to T cell-mediated immune responses and lead to resistance to immunotherapy [78].

### Cellular signaling pathways

The aberrant cellular signaling transduction is also a central factor contributing to the resistance to immunotherapy, such as PI3K/AKT pathway, WNT/β-catenin pathway, JAK/STAT/IFN-γ pathway, and mitogen-activated protein kinase (MAPK) pathway.

Oncogenic PI3K/AKT pathway has been proved to be associated with primary resistance to PD-1/PD-L1 checkpoint inhibition. PI3K/AKT signaling controls a variety of cellular processes including apoptosis, proliferation, motility, and metabolism, and contributes to tumor development and progression. Tumor suppressor PTEN, a lipid phosphatase, suppresses the activity of PI3K, and the loss of PTEN-mediated PI3K/AKT activation has been observed in many tumor types. In the Cancer Genome Atlas melanoma dataset, PTEN loss significantly correlated with the decreased expression of IFN-γ, granzyme B, less CD8 T cell infiltration, and further correlated with resistance to immune checkpoint therapy. In mice, the effectiveness of either PD-1/PD-L1 blockade or anti-CTLA4 mAb was enhanced by treatment with a selective PI3K inhibitor [79]. Whether the PI3K/AKT inhibitors would reverse the resistance to immune checkpoint blockade needs further clinical study.

In addition to the loss of PTEN, the potential of oncogenic signaling pathways to induce T cell exclusion from cancers has also been described through the stabilization of β-catenin resulting in constitutive WNT signaling pathway [80]. In a murine model, tumors with elevated β-catenin lacked a subset of CD103+ dendritic cells (DCs), due to decreased expression of CCL4, a chemokine that attracts CD103+ DCs. In addition, murine tumors lacking β-catenin responded effectively to immune checkpoint therapy whereas β-catenin-positive tumors did not. Non-T cell-inflamed human melanoma, which lacks T cells and CD103+ DCs in the tumor microenvironment, had significantly higher expression of tumor intrinsic β-catenin signaling genes. Therefore, cancer immune evasion and resistance to PD-1/PD-L1 blockade therapy could result from some crucial oncogenic signals, which might be new candidate targets for immune potentiation.

The IFN-γ pathway is emerging as a key player in primary, adaptive, and acquired resistance to checkpoint blockade therapy [81–83]. It has both favorable and detrimental effects on anti-tumor immune responses. Interferon-γ produced by tumor-specific T cells that have recognized their cognate antigen on cancer cells or APCs induces an effective anti-tumor immune response. However, continuous IFN-γ exposure can lead to immunoediting of cancer cells, resulting in immune escape [84, 85]. One mechanism by which cancer cells could escape the effects of IFN-γ is by decreasing the expression or mutations in molecules in IFN-γ signaling pathway, which goes through the IFN-γ receptors JAK1 and/or JAK2 and the signal transducer and activators of transcription (STATs) [86]. Analysis of tumors in patients who did not respond to therapy with the anti-CTLA-4 antibody ipilimumab revealed an enriched frequency of mutations in the IFN-γ pathway genes IFN-γ receptor 1 and 2 (IFNGR1 and IFNGR2), JAK2, and interferon regulatory factor 1 (IRF1) [81]. Any of these mutations would prevent signaling in response to IFN-γ and give an advantage to the tumor cells escaping from T cells, thereby resulting in primary resistance to anti-CTLA-4 therapy, and may also be a reason for the resistance to PD-1/PD-L1 blockade therapy. Mutations in this pathway would additionally result in lack of PD-L1 expression upon IFN-γ exposure, thereby resulting in cancer cells that would be genetically negative for inducible PD-L1 expression. In such a condition, blocking PD-L1 or PD-1 with therapeutic antibodies would not be useful, and these would be patients who are primarily resistant to PD-1/PD-L1 blockade therapy [87, 88].

The MAPK pathway plays an essential role in cell proliferation, and hyperactivation of MAPK signaling might also be related to the resistance to immunotherapy. With the inducing of VEGF and IL-8, MAPK signaling has inhibitory effects on T cell recruitment and function [89]. Inhibition of MAPK pathway promoted CD8+ T cell activation and infiltration, and induced the expression of tumor antigens as detected in human melanoma samples; in addition, acquired resistance to MAPK-targeted therapy was correlated with depletion of intratumor T cells, exhaustion of CD8 T cells, and loss of antigen presentation [90, 91]. Moreover, the combination of PD-1 blockade and short-term dual inhibition of BRAF and MEK could enhance tumor immune infiltration and improved tumor regression, suggesting that a nongenomic mechanism of MAPK inhibitor resistance might mediate cross-resistance to PD-1/PD-L1 blockade therapy [92].

### Tumor microenvironment

Tumor cells closely interact with the stromal cells, immune cells, and extracellular in the immunosuppressive TME, protecting tumor cells from being detected and eradicated by immunosurveillance [93]. Within the TME, other than tumor cells, components that might be associated with primary or acquired resistance include exhausted T cells, Tregs, myeloid derived suppressor cells (MDSCs), M2 macrophages, and other inhibitory immune checkpoints and cytokines.

T cell exhaustion is manifested by dysfunction, sustained expression of inhibitory receptors, and different transcriptional status with functional effector or memory T cells. Exhausted CD8 T cells with intermediate expression of PD-1 can benefit from PD-1/PD-L1 blockade, whereas the severely exhausted CD8 T cells with PD-1^high^ phenotype cannot benefit even impair the efficacy [94]. Thus, the ratio of partially exhausted PD-1^intermediate^ CD8+ T cells to severely exhausted PD-1^high^ CD8+ T cells might be a critical factor for the reversal of T cell exhaustion by PD-1/PD-L1 blockade.

Tregs are known to suppress effector T cell (Teff) responses by secretion of certain inhibitory cytokines, such as IL-10, IL-35, and transforming growth factor beta (TGF-β) [95–97]. *In vivo* studies have shown that depletion of Treg cells from the TME can strengthen the anti-tumor immune response [98–100]. Moreover, response to PD-1/PD-L1 blockade therapy was shown to be associated with increased ratio of Teff to Treg [101]. These data suggest that tumors without an increase of Teff and decrease of Treg following immunotherapy might be resistant to the treatment.

Myeloid-derived suppressor cells (MDSCs) have been considered as major regulators of immune responses in various pathological conditions. MDSCs could promote angiogenesis, tumor invasion, and metastasis [102]. Clinical findings have pointed out that the presence of MDSCs may be related to short survival in human cancers, such as breast and colorectal cancer [103]. Furthermore, the presence of MDSCs in TME contributed to decreased efficacy of immunotherapies, including immune checkpoint blockade [104]. In various tumor-bearing mice models, selective inhibition of MDSC by using PI3K inhibitors enhanced expression of proinflammatory cytokines and inhibited immunosuppressive factors, synergized with immune checkpoint inhibitors to promote tumor regression [105, 106]. These studies highlight inhibitors of PI3Ka as a potential therapeutic target for combination strategies with PD-1/PD-L1 blockade therapy.

Tumor-associated macrophages (TAMs) are another subset of cells that influence the responses to immunotherapy. TAMs include M1 macrophages, which are involved in promoting anti-tumor immunity, and M2 macrophages, which possess pro-tumorigenic properties [107]. Clinical studies have identified an association between higher frequencies of TAMs and poor prognosis in human cancers [108]. In a lung adenocarcinoma mice model, depletion of TAMs inhibited tumor growth as a result of decreased M2 TAM recruitment, possibly due to the inactivation of CCL2 and CCR2 signaling [109]. Reports suggest that macrophages can directly suppress T cell responses through PD-L1 in hepatocellular carcinoma [41]. To overcome the potential resistance of macrophages, blocking of CSF-1R, a receptor for macrophage colony-stimulating growth factor, in a pancreatic cancer-bearing mice model, decreased frequencies of TAMs, with subsequent increase in interferon production and tumor rejection. Importantly, CSF-1R blockade in combination with antibody against PD-1 or CTLA-4, in addition to gemcitabine, led to strengthened tumor regression [110].

Immunosuppressive cytokines are often released by tumor or macrophages for local suppression of anti-tumor immune responses. TGF-β plays an important role in immunosuppression by stimulating Tregs [111]. Increased TGF-β is associated with poor prognosis in multiple cancer types [112]. A preclinical study on radiation therapy combined with TGF-β inhibitor showed anti-tumor responses [113]. In addition, specific chemokines and chemokine receptors play a necessary role in trafficking of MDSCs and Tregs into tumors. For example, tumor secrete ligands CCL5, CCL7, and CXCL8, bind to their receptors CCR1 or CXCR2 expressed on subtypes of MDSCs, and attract MDSCs in the tumor microenvironment. Inhibitors of these chemokine receptors could abrogate immune evasion and improve anti-tumor T cell responses [114].

Indole 2,3-dioxygenase (IDO), which can be produced by tumors or immune cells, could improve the generation and activity of Tregs and MDSCs [115, 116]. IDO, as a rate-limiting enzyme, influences the catabolism of tryptophan, producing immunosuppressive metabolites to inhibit the proliferation of T cells and induce T cell anergy and apoptosis [117]. Holmgaard et al. found a marked delay in B16 melanoma tumor growth and increased overall survival in IDO knockout mice as compared with wild-type mice when treated with anti-CTLA-4/PD-1 antibody [118]. Based on the study, the combination of IDO inhibitors and PD-1/PD-L1 blockade may lead to more efficacious therapeutic anti-tumor immunity than applying the individual agent.

The immune response is dynamic, and the expression of immune molecules is fluctuated. Other than PD-1, overexpression of multiple inhibitory receptors such as T-cell immunoglobulin mucin 3 (TIM3), CTLA4, lymphocyte activation gene 3 (LAG3), and B and T lymphocyte attenuator (BTLA) is associated with inhibition of T-cell function and hamper to PD-1/PD-L1 blockade [119, 120]. Moreover, a recurrent tumor after PD-1/PD-L1 blockade treatment might be the result of increased expression of TIM-3 on T cells. Notably, preclinical studies showed that PD-1/PD-L1 blockade plus anti-TIM-3 led to improved responses in the tumor-bearing mice. Based on the fact that when one immune checkpoint is blocked, the other immune checkpoints may be induced, and hence, combination of PD-1/PD-L1 blockade with other immune checkpoint inhibitors may promisingly enhance antitumor responses.

### IPRES signatures

IPRES signatures, a set of 26 transcriptomic signatures, were found co-enriched to improve resistance to PD-1/PD-L1 blockade in tumors form nonresponding melanoma patients and the upexpression of IPRES related to the regulation of mesenchymal transition, cell adhesion, extracellular matrix (ECM) remodeling, angiogenesis and wound-healing [121]. The confirmation of IPRES co-enrichment in other independent tumor such as pancreatic adenocarcinoma indicates a transcriptomic program among different type of cancers and it may be a new way to enhance the efficacy of PD-1/PD-L1 blockade by inhibit the IPRES relevant biological processes.

### Epigenetic modification

Epigenetic modification in cancer cells may lead to changes in gene expression of immune-related genes, which can affect antigen processing, presentation, immune evasion, and T-cell exhaustion [122, 123]. Epigenetic modifying agents, DNA methyltransferase inhibitors and histone deacetylase inhibitors can reverse immune suppression via enhancing the expression of tumor-associated antigens, costimulatory molecules, components of antigen processing and presenting, other immune-related genes and chemokines [124, 125]. In addition, the low-dose DNA demethylating agent decitabine could directly promote T cell activation and cytolytic capacity with increased frequency of IFN-γ-expressing T cells [126]. Based on these findings, it is probably that epigenetic modification may ‘prime’ the host immune response for subsequent immunotherapy in combination therapy [124, 127, 128], and the efficacy of the combined strategy has been confirmed in both clinical studies and animal models [129–131]. Furthermore, immune priming by epigenetic therapy has been observed in combination with immune checkpoint inhibitors [132, 133]. Recently, it has been revealed that T cell exhaustion is associated with *de novo* DNA methylation, which can persist and be passed on to successive generations of T cells, whereas inhibition of DNA methylation by decitabine could reverse the exhausted state and promote T-cell rejuvenation [134]. These results establish a highly promising strategy for combination treatments by using epigenetic modifying agents and immune checkpoint blockade in cancer patients.

### Hyperaggressive disease with anti-PD-1/PD-L1 therapy

Besides primary and acquired resistance to immunotherapy, it was recently reported that PD-1/PD-L1 blockade might develop “hyperprogressive disease” in some patients, which means accelerated tumor growth after anti-PD-1/PD-L1 inhibitors [135]. A trial of 131 patients with PD-1 blockade therapies reported that 12 patients (9%) were considered as hyperprogressive disease; moreover, the hyperprogressive status seemed to be more common in elder patients over 65 year old [136]. Kurzrock et al reported that 6 patients out of 155 had extra copies of *MDM2* or *MDM4* genes and experienced time-to-treatment failure (TTF) of less than 2 months after immune checkpoint blockade therapy. Among these 6 patients, 4 patients developed hyperprogressive disease. In addition, patients with mutations in *EGFR* were also likely to experience short TTF and cause hyperprogressive disease [137]. Thus, exploring the mechanisms of resistance and hyperprogressive status to PD-1/PD-L1 blockade is particularly crucial. Actually, there is still not enough evidence to confirm that the accelerated tumor growth is pinned on immunotherapy, the hyperprogressive status could only occur with PD-1/PD-L1 blockade therapy in certain patients.

## PROSPECTIVE

PD-1 has now been proved to be an important checkpoint inhibitory receptor that impacts the T-cell stimulation, differentiation, and anti-tumor immune function. Both primary and acquired resistance to anti-PD-1/PD-L1 antibodies inspired us to investigate novel strategies to augment the efficiency of PD-1/PD-L1 blockade. First, radiotherapy, chemotherapy, epigenetic therapy, and other immune stimulatory agents combined with PD-1/PD-L1 blockade could enhance the sensitivity of immunotherapy in tumors with low immunogenicity. Second, modulating the immunosuppressive tumor microenvironment and breaking the inhibitory status, such as depletion of Tregs, interference with the suppressive cytokines, and silencing of co-inhibitory receptors could also help increase the therapeutic responses of PD-1/PD-L1 blockade. Third, further understanding and exploring the mechanisms of both the upstream regulators of PD-1 and its downstream biological events and targets will be necessary for the design of combination therapies, to illuminate the resistance mechanisms, and identify the reliable predictive biomarkers of PD-1/PD-L1 blockade. Last, with the progress made in human genome sequencing and bioinformation analysis, detection and understanding of the patients' genetic and epigenetic information of tumors and immunocytes will help establish individualized immunotherapy, to obtain clinical benefits for more patients.
